# Biosynthesis of silver nanoparticles from *Schizophyllum radiatum* HE 863742.1: their characterization and antimicrobial activity

**DOI:** 10.1007/s13205-013-0138-0

**Published:** 2013-06-09

**Authors:** Ram Prasad Metuku, Shivakrishna Pabba, Samatha Burra, S. V. S. S. S. L. Hima Bindu N, Krishna Gudikandula, M. A. Singara Charya

**Affiliations:** Department of Microbiology, Kakatiya University, Hanamkonda, Warangal, 506009 India

**Keywords:** White rot fungi, Silver nanoparticles, Antimicrobial activity, Scanning electron microscopy

## Abstract

Development of reliable and eco-friendly process for synthesis of silver nanoparticles is an important step in the field of application in nanotechnology. One of the options to achieve this objective is to use natural biological processes. They have an advantage over conventional methods involving chemical agents associated with environmental toxicity. This study demonstrates the extra-cellular synthesis of stable silver nanoparticles using the white rot fungus, *Schizophyllum radiatum* with GenBank Accession no HE 863742.1. The supernatant of the seed media obtained after separating the cells has been used for the synthesis of silver nanoparticles. The morphology and structure of synthesized silver nanoparticles were characterized using FT-IR, XRD, UV–visible spectrum of the aqueous medium containing silver ion showed a peak in the range of 420–430 nm corresponding to the Plasmon absorbance of silver nanoparticles. Scanning electron microscopy micrograph showed formation of well-dispersed silver nanoparticles in the range of 10–40 nm. The effect of different carbon sources and the time taken for formation particles and the anti-microbial activity of synthesized nanoparticles were carried and compared with silver nitrate solution and with standard streptomycin. The process of reduction being extra-cellular and fast may lead to the development of an easy bioprocess for synthesis of silver nanoparticles.

## Introduction

Nanobiotechnology attempts to utilize biological templates in the development of nano-scaled products for diverse and specialized applications. Nanoparticles are clusters of atoms in the size range of 1–100 nm. Increasing concern in green chemistry approaches for nanomaterial synthesis and process technology development has provided additional impetus for bioprocess studies using both prokaryotic bacterial cells and eukaryotic organisms (Bhattacharya and Gupta 2005). The size, shape and intercalation properties are the attributes of nanomaterials. The whole cell and cellular biomolecules have evolved as one of the best ways for generation and functionalization in the nanocomposite (Agag and Takeichi 2000). Biological organisms can be used as the environmental friendly techniques to create predictable nanoparticles. Silver and gold nanoparticles synthesized extra-cellularly that have the potentials in the opto electronic devices, thin films and non linear optics (Dahl et al. 2007). Macromolecules like enzymes and polysaccharides from the bioprocesses are increasingly in focus for nanomaterial production and utilization. Bacteriorhodopsin is one of the extensively studied nanomaterials for technical application, including in photoelectric and proton transport devices (Hampp 2004). Silver nanoparticles are in demand for the photo chemical applications, catalysis, and chemical analysis. The microbial silver bio-inorganics were studied intensively (Lengke et al. 2007; Shiying et al. 2007; Morones et al. 2005). The applications of AgNPs are of great concern in waste water treatment, pesticide degradation, killing human pathogenic bacteria (Kuber and Souza 2006). They exhibited cyto-protectivity toward HIV-1 infected cells (Elechiguerra et al. 2005). The study of biosynthesis of nanomaterials offers a valuable contribution as eco-friendly technologies into material chemistry. The ability of some microorganisms such as bacteria and fungi to control the synthesis of metallic nanoparticles should be employed in the search for new materials (Mandal et al. 2006).

This study was on the potentials of extra-cellular biosynthesis of silver nanoparticles by *Schizophyllum radiatum* and their characterization, antimicrobial activity on Gram-positive and Gram-negative bacteria. The influence of carbon sources on the silver nanoparticles biosynthesis was carried out. The *S. radiatum* was also studied for its antimicrobial and antioxidative properties under submerged fermentation. The supernatant of the seed media obtained after separating the mycelia has been used for the synthesis of silver nanoparticles.

## Materials and methods

### Chemicals

Dextrose, silver nitrate Merck (Germany) Malt extract was procured from Himedia (India). Sterile distilled water was used throughout the experiments.

### Collection and molecular identification of *Schizophyllum radiatum*

Fungi in the form of fruit body were collected from the Eturnagaram forest of Warangal, Andhra Pradesh, India. The fruit body was cleaned with disinfectants and approximately 3 × 3 mm was placed on MEA agar medium in petri-dishes. When the mycelium had grown on the medium in the vicinity of the tissues, the sample was transferred to fresh agar media in tubes. This was repeatedly carried out until pure culture was obtained. Molecular-based characterization on ribotyping of 18S rRNA was performed at the Xcelris Genomics, Ahmedabad, India and sequence was deposited to EMBL database for accession number.

### Production of extra-cellular silver nanoparticles

*Schizophyllum radiatum* was grown in yeast malt broth containing dextrose 10 g l^−1^, malt extract 5 g l^−1^. The final pH was adjusted to 6.0. The flasks were incubated in the orbital shaker at 200 rpm at 32 °C. After 5 days of incubation, the mycelium was separated by filtration and supernatant was challenged with equal amount of with various concentrations (0.5, 1.0, 1.5, 2.0, 2.5 mM) of silver nitrate solution (prepared in deionized water) and incubated in shaker at 200 rpm in dark condition at 32 °C. Simultaneously, a positive control of silver nitrate solution and deionized water and a negative control containing only silver nitrate solution were maintained under same conditions.

### Influence of carbon sources on silver nanoparticle synthesis

In order to investigate the influence of carbon sources on the silver nanoparticle synthesis, the time taken for the formation of particles (i.e. from colorless to brown) was studied. Sources like glucose, fructose, sucrose, lactose, and starch were added separately in place of dextrose at a concentration of 0.4 % to the basal medium containing malt extract (1 %). *S. radiatum* was inoculated into the basal medium containing particular carbon source and incubated at 32 °C for 5 days at 200 rpm in orbital shaker. After incubation, the culture broth was filtered and separated the mycelial biomass. Supernatant was challenged with equal volume of effective concentration of AgNO_3_ solution. Control consisting of filtered broth of particular carbon source and deionized water was maintained under same conditions.

### UV–visible spectral analysis

Change in color was observed in the silver nitrate solution incubated with the *S. radiatum*. The UV–visible spectra of this solution were recorded in ELICO SL-159 Spectrophotometer in the range of 350–470 nm.

### Scanning electron microscope

After freeze drying of the purified silver particles, the size and shape were analyzed by scanning electron microscopy (JOEL-Model 6390).

### Fourier-transform infrared (FT-IR) chemical analysis

Fourier-Transform Infra-Red spectroscopy measurements, the biotransformed products present in extracellular filtrate were freeze-dried and diluted with potassium bromide in the ratio of 1:100. The FT-IR spectrum of samples was recorded on a FT-IR instrument (Digital Excalibur 3000 series, Japan) with diffuse reflectance mode (DRS-800) attachment. All measurements were carried out in the range of 400–4,000 cm^−1^ at a resolution of 4 cm^−1^ (Saifuddin et al. 2009).

### X-ray diffraction analysis

The fungal mycelium embedded with the silver nanoparticles was freeze-dried, powdered and used for XRD analysis. The spectra were recorded in Philips^®^ automatic X-ray Diffractometer with Philips^®^ PW 1830 X-ray generator. The diffracted intensities were recorded from 30° to 90° 2*θ* angles.

### Antibacterial activity

Biosynthesized silver nanoparticles produced by the *S. radiatum* were tested for antimicrobial activity as method suggested by Srinivasulu et al. (2002) using various Gram-positive and Gram-negative bacteria by the agar well-diffusion method. Approximately, 20 ml of nutrient agar medium was poured into sterilized petri-dishes. The bacterial test organisms were grown in nutrient broth for 24 h. A 100 μl nutrient broth culture of each bacterial organism (1 × 10^5^ CFU ml^−1^) was used to prepare bacterial lawns. Agar wells of 8 mm diameter were prepared with the help of a sterilized stainless steel cork borer. The wells were loaded with 60 μl of Ag nanoparticles solution, 60 μl of 1.5 mM silver nitrate and 60 μl of culture broth from *S. radiatum* culture without AgNO_3_ as a negative control, along with 60 μl of 30 μg ml^−1^ of streptomycin as a positive control. The plates were incubated at 37 °C for 24 h and then were examined for the presence of zones of inhibition. The diameter of such zones of inhibition was measured and the mean value for each organism was recorded and expressed in millimeter unit.

## Results and discussions

The 18S rRNA gene sequencing analysis of the isolate yielded 1,112 base pairs, and NCBI BLAST search analysis based on the topology of phylogenetic analysis revealed that the sequence was 99 % related with *S. radiatum.* The obtained sequence was deposited in EMBL database with the Accession number HE 863742.1.

### Biosynthesis of silver nanoparticles

**Fig. 1:**
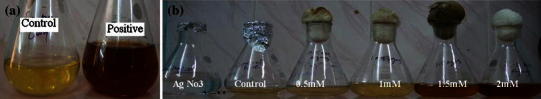
**a** Showing the formation of silver nanoparticles after 48 h of incubation. **b** Effect of various concentrations of silver nitrate AgNO_3_ on nanoparticle synthesis

**Fig. 2:**
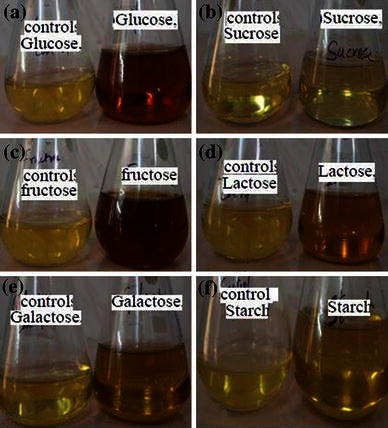
Influence of carbon sources on nanoparticles synthesis **a** glucose, **b** sucrose, **c** fructose, **d** lactose, **e** galactose, **f** starch with respective controls

Bioreduction of silver nitrate into nanosilver can be primarily characterized using UV–vis spectroscopy. In the present study, the color of the silver nitrate solution in the flask containing culture filtrate of *S. radiatum* changed from colorless to brown within 48 h. Various concentrations of silver nitrate (0.5, 1.0, 1.5, 2.0 and 2.5 mM) that were used, the formation of silver nanoparticles synthesis was observed at 1.5 mM within 48 h (Fig. 1) and further increased with increase in silver nitrate concentration. An appreciable amount of silver nanoparticle was obtained at 2.5 mM concentration. Kathiresan et al. (2009), working with silver nanoparticles in the range of 400–500 nm wave length, reported that there is a decrease in the value of optical density (OD) with a proportional increase in silver nitrate concentration. Extracts from organisms may act as reducing and capping agents in AgNPs synthesis. The reduction of silver ions by combinations of bio-molecules found in these extracts such as enzymes, proteins, amino acids, polysaccharides and vitamins is environmentally benign, yet chemically complex. But, the mechanism which is widely accepted for the synthesis of silver nanoparticles is the presence of enzyme “Nitrate reductase” (Anil Kumar et al. 2007; Kalimuthu et al. 2008). Cysteine a biomolecule present in the cell-free extract from *Trichoderma asperellum* acts as a potential reducing and capping agent in the synthesis of stable silver nanoparticles (Roy et al. 2011). From application point of view, the extracellular synthesis of nanoparticles is more important than the intracellular assimilations. Carbon sources such as glucose and fructose enhanced the production of enzyme which in turn reduced the silver nitrate hence the amounts of silver nanoparticles were improved. The time taken for the formation of silver nanoparticles appeared with in 48 h in glucose- and fructose-containing carbon sources. Lactose and galactose showed 72 h, whereas starch has no influence on silver nanoparticle synthesis (Fig. 2).

### Characterization of silver nanoparticles

**Fig. 3:**
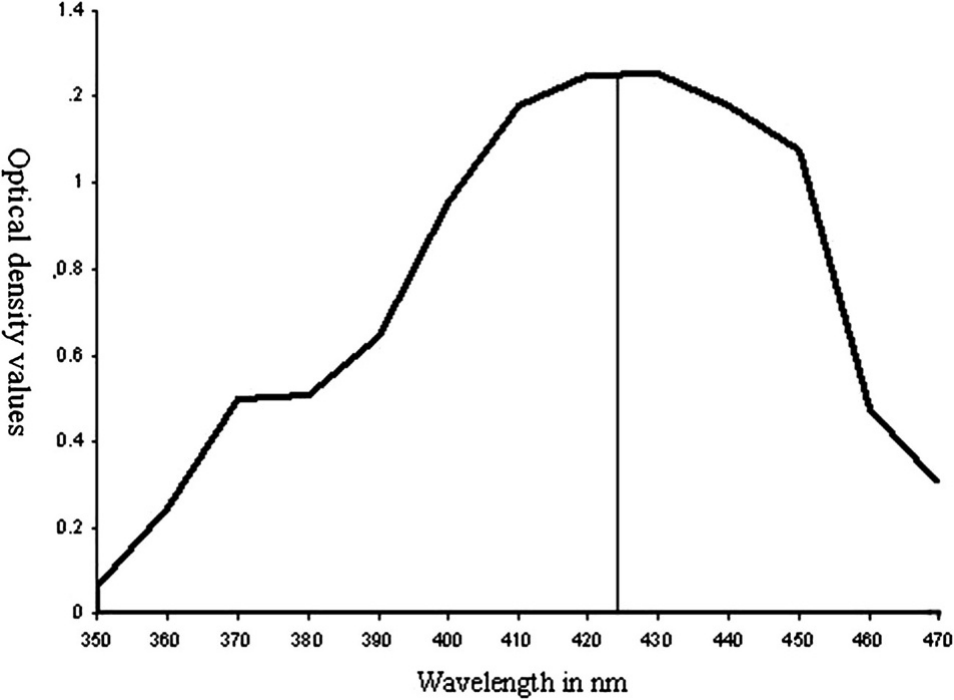
UV–visible absorption spectra of silver nanoparticles after 48 h of incubation

The homogeneous spherical silver nanoparticles are known to produce the surface plasmon resonance band at 413 nm (Ahmad et al. 2003). In the present system absorption maxima (*λ*_max_) of biosynthesized silver nanoparticles (Fig. 3) observed in the range of 420–430 nm. Brause et al. (2002), working with silver colloids in aqueous solution, reported that optical absorption spectra of metal nanoparticles are mainly dominated by surface plasmon resonance, and the absorption peak has relationship with particle size. Smaller AgNPs will have an absorbance maximum around 400 nm, which increases with size and disappears when particle size falls outside nanodimensions.

### Scanning electron microscopy (SEM) analysis

**Fig. 4:**
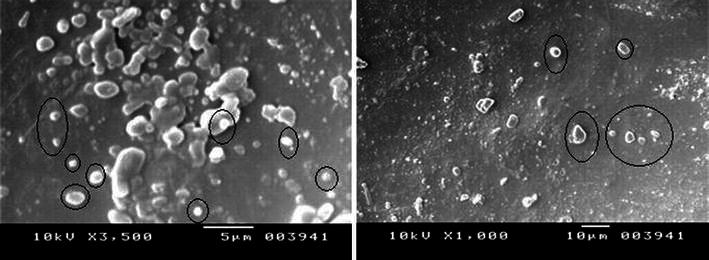
Shows SEM micrographs of silver nanoparticles synthesized from fungal extracts

This SEM image (Fig. 4) provided further insight into the morphology and size of the produced nanoparticles. It is evident from the figure that the biosynthesized silver nanoparticles are in different size and shapes and mostly observed as individual particles as well as a few aggregates. SEM-mediated characterization of biosynthesized nanomaterials has been performed by several investigators. Particle size analysis revealed that the silver nanoparticles are in the size range of 10–40 nm with a mean diameter of 14.5 nm suggesting the production of different-sized nanoparticles. Silver nanoparticles in the range of 35–46 nm by *Pseudomonas stutzeri* (Klaus et al. 1999) 20–50 nm particles by *Lactobacillus* sp. (Nair and Pradeep 2002) 2–20 nm particles by *Verticillium* sp. and 2–50 nm sized particles by *Fussarium oxysporum* (Ahmad et al. 2003) have been reported. Mukherjee et al. (2008) demonstrated the synthesis of low dispersive and highly stabilized nanocrystalline silver particles by a non-pathogenic and agriculturally important fungus, *Trichoderma asperellum*.

### Fourier-transform infrared (FT-IR) chemical analysis

**Fig. 5:**
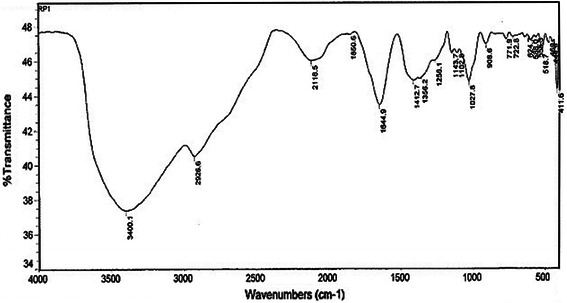
FT-IR spectrum recorded with synthesized silver nanoparticles

FT-IR spectroscopy, used to characterize the surface chemistry of silver nanoparticles produced by *S. radiatum* (Fig. 5), showed the FT-IR spectrum of the freeze-dried powder of silver nanoparticles formed after 48 h of reaction. The spectral data revealed two types of vibrations (i.e. stretching and bending) in the wavelength range of 4,000–500 cm^−1^. It is evident from the figure that the presence of an amine vibration band at 3,400 cm^−1^ represents a primary amine (N–H) stretching, and amide (N–H) bending vibration bands at 1,650 and 1,644 cm^−1^. Furthermore, the FT-IR spectra of biosynthesized silver nanoparticles also revealed peaks at 2,026 and 2,116 cm^−1^ stretching vibrations of aliphatic C–H bonds. A band presence at 1,412 cm^−1^ can be assigned to CH2-scissoring stretching vibration at the planar region. Several C–N stretching vibration peaks at 1,258, 1,143, 1,102, 1,027 and 908 cm^−1^ were also observed in the spectral range of 1,230–900 cm^−1^. In addition, the presence of bands at 1,356 and 1,250 cm^−1^ in the FT-IR spectra suggested that the capping agent of biosynthesized nanoparticles possesses an aromatic amine groups with specific signatures of amide linkages between amino acid residues in the proteins in the infrared region of the electromagnetic spectrum (Shaligram et al. 2009). This type of FT-IR spectra supports the presence of a protein type of compound on the surface of biosynthesized nanoparticles, confirming that metabolically produced proteins acted as capping agents during production and prevented the reduced silver particles agglomeration.

### X-ray diffraction analysis

**Fig. 6:**
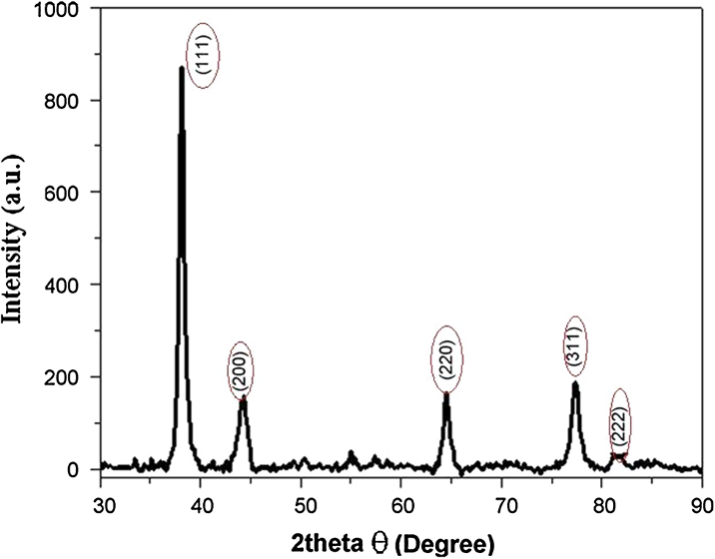
XRD pattern of silver nanoparticles

Freeze-dried reaction mixture embedded with the silver nanoparticles was used for X-ray diffraction (XRD) analysis. Crystallinity of biosynthesized silver nanoparticles was assessed from their X-ray powder diffraction patterns. The diffractogram showed (Fig. 6) the phase purity of the material. The diffraction peaks are above 37°, XRD patterns were recorded the four prominent 111, 200, 220 and 311 reflections at 2*θ* = 38.2, 44.4, 64.5 and 77.7 indicating the face centered cubic (FCC) structure of silver nanoparticles.

### Anti bacterial activity

**Fig. 7:**
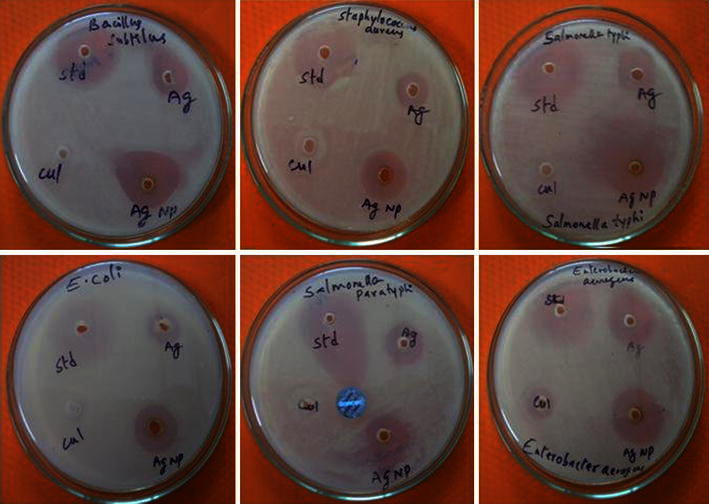
Antibacterial activity of silver nanoparticles produced by *Schizophyllum radiatum* against bacterial species, where *std* standard (streptomycin), *cul* culture broth, *Ag* silver nitrate solution, *AgNP* silver nanoparticles solution

Silver has been in use since time immemorial in the form of metallic silver, silver nitrate, silver sulfadiazine for the treatment of burns, wounds and several bacterial infections. But due to the emergence of several antibiotics the use of these silver compounds has been declined remarkably. Silver in the form of silver nanoparticles has made a remarkable comeback as a potential antimicrobial agent and proved to be most effective as it has good antimicrobial efficacy against bacteria, viruses and other eukaryotic micro-organisms (Gong et al. 2007). The use of silver nanoparticles is also important, as several pathogenic bacteria have developed resistance against various antibiotics. The antibacterial activity of silver nanoparticles was investigated against various pathogenic Gram-positive and Gram-negative bacteria, like *Escherichia coli*, *Klebsiella pneumoniae*, *Enterobacter aerogenes*, *Pseudomonas aeroginosa*, *Staphylococcus aureus*, *Salmonella paratyphi*, *Bacillus sterothermophilus*, *Bacillus subtilus* using well-diffusion technique (Fig. 7). The diameter of inhibition zones around each well with AgNPs and AgNO_3_ is recorded in Table 1. The highest antimicrobial activity was observed against, *B. subtilus* and *S.paratyphi,* followed by *Bacillus stearothermophilus*, *Staphylococcus aureus*, *Enterobacter aerogenes*, *Salmonella typhi* and the least was noticed against *Klebsiella pneumonia.* Feng et al. (2000) reported the mechanism of silver nanoparticles action and used *E. coli* and *S. aureus* as model organisms. The nanoparticles get attached to the cell membrane and also penetrate inside the bacteria. The bacterial membrane contains sulfur-containing proteins and the silver nanoparticles interact with these proteins in the cell as well as with the phosphorus-containing compounds like DNA. Nanoparticles preferably attack the respiratory chain, cell division finally leading to cell death. Silver nanoparticles have emerged up with diverse medical applications in silver-based dressings (Duran et al. 2007).

**Table 1:** Antibacterial activity of silver nanoparticles produced by *Schizophyllum radiatum*

Microorganism	Zone of inhibition (mm)		
	Streptomycin^a^	AgNO_3_ sol^b^	Silver nanoparticle sol^c^
*Bacillus subtilus*	20	9	19
*B. stearothermophilus*	24	13	17
*Salmonella paratyphi*	24	8	19
*S. typhi*	23	9	14
*Staphylococcus aureus*	22	8	15
*Pseudomonas aeroginosa*	23	9	13
*Enterobacter aerogenes*	23	16	16
*E. coli*	24	6	14
*Klebsiella pneumoniae*	14	3	7

^a^Zone of growth inhibition diameter of streptomycin (positive control)

^b^Zone of growth inhibition diameter with silver nitrate sol

^c^Zone of growth inhibition diameter with silver nanoparticles

## Conclusion

The nanoscale understanding of the bioprocesses and interventions are only in infancy. The areas particularly bioprocess product exploitations are only in current focus that in itself has opened vistas in a technology upsurge. The order beneath the chaos at the genomic level is recognizable but not yet been fully understood. The microorganisms such as bacteria and algae have already proved to be inorganic nanofactories of the enormous dimensions. Among the different methods for NP synthesis, the chemical reduction method and green synthesis method were widely studied due to their advantage in controlling particle size and morphology. White rot fungus, easy to produce biomass and non-pathogenic nature will add strength to silver nanoparticles biosynthesis.
